# Report of the Chairman of the Committee on Intelligence and Education1Read before the Annual Convention, U. S. V. M. A., Des Moines, Iowa, September, 1895.

**Published:** 1896-02

**Authors:** C. A. Cary


### REPORT OF THE CHAIRMAN OF THE COMMITTEE ON INTELLIGENCE AND EDUCATION.[1]

As a member of the committee there are some living issues that are of vital importance which I wish to review hurriedly.

During the past year one State school and one private institution have passed into history. While we may regret the passing of the one, we cannot mourn over the loss of any little private affair. No strictly private veterinary college in Europe or America has succeeded, and I doubt if any patronage school ever will erect anything like a complete veterinary institution. The writer still maintains that veterinary education, which so vitally affects wealth and public health, should be entirely under the control of the State.

The United States is still blessed with a superabundance of veterinary colleges. One in Iowa, one in Illinois, one in Ohio, one in Pennsylvania, one in Massachusetts, two in New York State, and one for the entire South would more than supply the demand. It seems that all the small men in the profession have gone wild on the necessity of having a place in a college. Make a list of the men on the teaching staff of all the veterinary colleges of America. Now strike out the names of those who are partially or wholly unfitted by education, experience and teaching ability, and it will be seen that over one-half of the total number are men who could not by merit, by natural ability, or by a competitive examination secure the positions they now fill. The faculties of nearly all of the private schools are made up of local men who will work for prestige—a kind of practice prestige.

The question suggests itself as to whether a man can conduct a large practice and at the same time be an efficient instructor or an investigator in a college. A position in the faculty of a private veterinary college may help a man's private practice, but he will certainly fail to elevate the standing and efficiency of the college.

Surprising to most men in the profession, one of the smaller two-year course private colleges now offers a post-graduate course. Is it prepared to meet the demands of post graduates?

[1] Read before the Annual Convention, U. S.V. M. A., Des Moines, Iowa, September, 1895.

If so, let the good work go on. There should be a demand for post-graduate work, for observation and contact with men in practice inform us that there are practitioners sorely in need of post-graduate instruction. A summer post-graduate course by the Veterinary Department of the University of Pennsylvania, or by Cornell as she is to be, would certainly be in order.

During the past two years I have from necessity read much in the catalogues of veterinary colleges. They are so much alike that it must puzzle the young men who peruse them to determine where they shall go to buy their veterinary knowledge. Above all, a college catalogue should give a tabulated statement of the courses of study or lectures. These tables should state the names of the subjects taught, the number of hours daily and weekly devoted to each subject, and how many months each subject is considered. This can be easily done. One can then make an intelligent comparison of the course of lectures in one college with another, or with several others. As the catalogues are now issued it is almost impossible to make a table that would enable this Association to compare the different college lecture courses with one another.

There is some question as to the degree of honesty or integrity in the open and bold announcements and statements made in some of the veterinary college catalogues. For example:

One catalogue states that "the college hospital is one of the finest, best equipped and ventilated in the world."

Another states that "each student will be given every facility to conduct experiments (in chemistry) under the direct supervision of the instructor."

Another catalogue states that the department of chemistry "embraces physics, organic and inorganic chemistry, and toxicology. Each of these branches is abundantly illustrated by the necessary apparatus and experiments, which are performed before the class." This institution has, in fact, a small chemical laboratory, with insufficient apparatus to make up the "necessary apparatus."

The Toronto Veterinary College says: "We deem it unnecessary to give an extended and complicated description of the synopsis of lectures, more likely to mystify than enlighten intending applicants. We merely state that the subjects taught are the same as in the modern European colleges, and all the lectures are delivered specially to veterinary students, the same as in London, Edinburgh, and Paris." How clear and how free from mystification! And, above all, how far from the facts or the truth are the assertions!

I could multiply quotations from catalogues, showing case after case of entire or partial prevarication or the grossest mystification. Most of these "deviations from the line of moral rectitude" are found in careless and exaggerated statements about what the college can do, its laboratory supplies and capacity, and its comparatively great equipment, buildings, and faculty.

In these extracts I have not sought to place all the most questionable statements before you, but I wish to call your attention to the fact that any and all institutions recognized by this Association should be required to announce the truth in no mystifying form, or be called to account for misstatements. We require the members of this Association to abide by a code of morals. Why should not the institutions which have much more to do with the formation of good moral principles in the majority of the men who make up the profession—why should they not be called upon to advertise honestly?

There is a growing demand for accurate and reliable records in the field of veterinary medicine and surgery. None of our American colleges publish annual statements in their respective catalogues or by special issue, giving a classified list of the clinical cases, of the surgical operations, of the post-mortem examinations, of the number of dissections, and of the cases of internal medicine. Special and full reports should be made of all peculiar cases, of discoveries of any value in pathology, surgery, or internal medicine. If the fountains of learning fail to be scientific, what must we expect of our offspring? I would also urge that each practitioner record every one of his cases and annually report them in some of the medical journals. No complete science will ever be built upon incomplete or unreliable records.

Lastly, I suggest that it is now time for this Association to give some recognition to the agricultural colleges that are so ably supplementing and advancing the veterinary education in the United States. At least a list of the recognized agricultural colleges should be published. A four years' course in the A. and M. colleges that have qualified veterinarians and suitable courses of study should admit a person to the second year in all our recognized three-year course veterinary colleges.

C. A. CARY.

